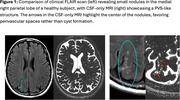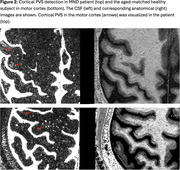# Exploring cortical perivascular space as a biomarker in neurodegenerative diseases through optimized CSF‐only MRI at 7T

**DOI:** 10.1002/alz.095695

**Published:** 2025-01-09

**Authors:** Zeynep Demir, Gael Saib, Lalith Talagala, Paul Taylor, Justin Y Kwan, Alan Koretsky

**Affiliations:** ^1^ National Institutes of Health, Bethesda, MD USA; ^2^ National Institute of Neurological Disorders and Stroke, Bethesda, MD USA

## Abstract

**Background:**

Detecting changes in perivascular spaces (PVS) holds promise as a biomarker for neurodegenerative diseases. These spaces exhibit increased protein accumulation and dilatation in neurodegenerative diseases even preceding symptomatic stages. Advanced MRI techniques at high fields offer unparalleled clarity in visualizing these subtle structures. Notably, patients with Alzheimer’s disease (AD) show increased number of dilated PVS compared to healthy individuals in 7T MRI. While previous studies have primarily focused on white matter PVS due to their large size and visibility, cortical PVS remain relatively unexplored despite their potential diagnostic significance in diseases such as AD and motor neuron disease (MND). Recently, we optimized heavily T2‐weighted Turbo Spin Echo (TSE) at 7T, exclusively highlighting cerebrospinal fluid (CSF) signals, thus enabling the visualization of cortical PVS for the first time in healthy individuals. Leveraging this ability to detect cortical PVS, we aim to investigate cortical PVS in neurodegenerative diseases.

**Method:**

Seven MND and 5 AD/ADRD patients enrolled in an NIH IRB approved protocol (NCT03225144), and 20 healthy controls were scanned using Seimens Terra 7T MRI with our optimized T2‐weighted TSE sequence (TR = 2430 ms, TE = 500 ms, ETL = 108, TA = 10 min) achieving an isotropic resolution of 0.5 mm (125 nl voxels).

**Result:**

The optimized sequence achieved a CSF‐to‐tissue signal ratio of approximately 30:1. After thresholding above noise, it was possible to detect CSF volumes of 10 nanoliters per voxel. In controls, cortical perivascular spaces were detected with unique morphology and have a density of 1‐2% of grey matter with the highest density in insular region. In one control, the CSF images revealed extensive PVS‐like structures in a previously suspected cyst formation at the juxtacortical area (Figure 1). Furthermore, patients with MND exhibited multiple distinct cortical PVS, most pronounced in the motor cortex (Figure 2).

**Conclusion:**

Our findings underscore the utility of CSF‐only MRI in uncovering intricate structural details of cortical PVS, which can be explored in neurodegenerative diseases. This non‐invasive imaging modality holds promise as a sensitive biomarker for neurodegenerative diseases, with ongoing efforts aimed at quantifying disease‐specific cortical PVS and elucidating clinical implications of changes on cortical PVS.